# Management of Combined Cardiac Surgery Using Cardiopulmonary Bypass With Acute Normovolemic Hemodilution in a Jehovah’s Witness: A Case Report

**DOI:** 10.7759/cureus.33442

**Published:** 2023-01-06

**Authors:** Takayuki Morimoto, Taiga Ichinomiya, Hiroaki Murata, Motohiro Sekino, Tetsuya Hara

**Affiliations:** 1 Department of Anesthesiology and Intensive Care Medicine, Nagasaki University Graduate School of Biomedical Sciences, Nagasaki, JPN

**Keywords:** preoperative hematopoiesis, cardiopulmonary bypass, combined cardiac surgery, acute normovolemic hemodilution, jehovah’s witness

## Abstract

Combined cardiac surgery under cardiopulmonary bypass (CPB) has a high risk of requiring blood transfusion. Performing this surgery on Jehovah's Witnesses (JWs) is challenging as they strictly refuse allogeneic blood transfusions due to their religious beliefs.

A 73-year-old female JW patient underwent combined surgery involving coronary artery bypass grafting and mitral valvuloplasty under CPB. Preoperative hematopoiesis maintained the hemoglobin (Hb) level at >12 g/dL preoperatively; the Hb level was maintained at >7 g/dL during CPB for effective acute normovolemic hemodilution (ANH). Compared with the values obtained immediately after CPB weaning, the Hb level and coagulation functions (measured using viscoelastic tests) improved after autologous transfusion at the end of the surgery.

When cardiac surgery under CPB is performed on JWs, ANH can be useful for maintaining postoperative Hb levels and coagulation factors. Sufficient preoperative hematopoiesis and determination of an appropriate volume for intraoperative ANH may be important for effective ANH.

## Introduction

Combined cardiac surgery under prolonged cardiopulmonary bypass (CPB) involves a high risk of requiring blood transfusion due to exacerbated bleeding and coagulopathy [1]. Jehovah’s Witnesses (JWs) refuse allogeneic blood transfusions due to their religious beliefs; thus, performing this complex procedure on them is especially challenging.

Recommended perioperative blood management protocols include increasing the hemoglobin (Hb) level preoperatively, achieving blood conservation with adjunctive hemostasis intraoperatively, and managing vitals postoperatively [2]. Pre-CPB acute normovolemic hemodilution (ANH) is effective for intraoperative blood conservation because it preserves the Hb, platelets, and coagulation factors consumed or diluted due to CPB [3]. Herein, we have described the successful case of a JW patient who underwent combined cardiac surgery under CPB following ANH. Preoperative hematopoiesis and determination of the appropriate ANH volume required (while considering the minimum Hb levels acceptable during CPB) may be important for effective ANH.

## Case presentation

A 73-year-old female JW patient (152 cm, 41 kg) was scheduled for a combined coronary artery bypass grafting (CABG) for three-vessel disease and mitral valvuloplasty (MVP) for severe mitral valve regurgitation. At presentation, laboratory data revealed mild anemia (Hb, 10.6 g/dL); however, coagulopathy was not observed (prothrombin time and international normalized ratio (PT-INR), 1.01; activated partial thromboplastin time (aPTT), 25 s; fibrinogen, 385 mg/dL; and platelets, 31.6×10^4^/μL). Due to her religious beliefs, the patient refused allogeneic transfusions and preoperative stored autologous blood but agreed to receive ANH, intraoperative blood salvaging autotransfusion, albumin, and fibrinogen concentrate. During a multidisciplinary discussion, pre- or intraoperative percutaneous coronary intervention (PCI) combined with MVP was proposed as an alternative to shorten the CPB duration and decrease blood loss. However, it was not adopted due to the difficulty of the PCI procedure and the risk of bleeding secondary to antiplatelet therapy. The hematopoietic intervention was initiated with intravenous erythropoietin (6,000 U, thrice a week) and oral iron (100 mg/day) and vitamin B12 (0.75 mg/day) supplementation. On the day of the surgery (i.e., after three weeks), the Hb level was 14 g/dL.

General anesthesia was maintained with sevoflurane and remifentanil before and after CPB, and with remimazolam and remifentanil during CPB. Standard coagulation and viscoelastic tests were performed using ClotPro® (enicor GmbH, Munich, Germany) to assess the coagulation functions intraoperatively. The maximum ANH volume was calculated as 400 mL using the following formula, assuming that the estimated circulating blood volume (EBV) was approximately 2.5 L (body weight × 60) [4], the minimum priming volume for the CPB circuit was 1,100 mL, and the acceptable Hb level was >7.5-8.0 g/dL after CPB induction.

$\frac{Preoperative\, Hb\, levels\, \times\, \left ( EBV\, -\, ANH\, volume \right ) }{EBV\, +\, Priming\, volume}\, > 7.5-8.0\, g/dL$

According to this formula, 400 mL autologous blood for ANH was drawn from the right internal jugular vein using the distal root of a triple central venous catheter after anesthesia induction. In contrast, the blood pressure was maintained with noradrenaline instead of volume loading to avoid hemodilution. The drawn autologous blood was kept connected to the central venous catheter. The Hb level after drawing autologous blood was 11.5 g/dL.

**Figure 1:**
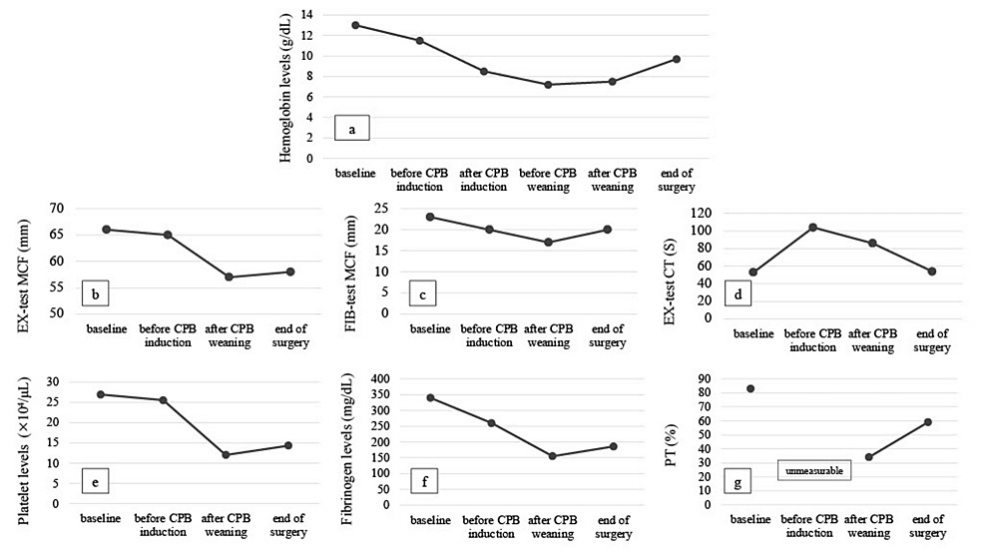
Intraoperative hemoglobin, platelet, and fibrinogen levels; EX-test MCF and CT; FIB-test MCF; and PT. The times shown are after anesthesia induction (baseline), after drawing autologous blood (before CPB induction), before administration of terminal warm cardioplegia (before CPB weaning), after CPB weaning with protamine (after CPB weaning), and after returning autologous blood (end of surgery). MCF, maximum clot firmness; CT, clotting time; PT, prothrombin time; CPB, cardiopulmonary bypass

Surgery was performed via median sternotomy; CPB was established with a drainage cannula into the superior and inferior vena cava and with a reinjection cannula into the ascending aorta. After harvesting grafts from the bilateral saphenous veins and left internal thoracic artery, CABG and MVP were performed with cardiac arrest under CPB (flow rates, 2.4-2.6 L/min/m^2^; perfusion pressure, 50-70 mmHg; and minimum rectal temperature, 34℃). Heparin was initially administered at 250 units/kg, and additional doses were administered thereafter to maintain an activated clotting time (ACT) of >400 s. Tranexamic acid (1,500 mg) was administered intermittently during the surgery. After CPB, the effect of heparin was antagonized by protamine. When the ACT decreased to 127 s, we began to return the full volume (400 mL) of autologous blood and administered 186 mL of blood salvage autotransfusion. The lowest Hb level recorded was 7.2 g/dL (immediately before CPB weaning); however, it increased to 9.7 g/dL at the end of the surgery (Figure 1a). The maximum clot firmness (EX-test and FIB-test), platelet level, and fibrinogen level were the lowest immediately after CPB weaning; these parameters did not return to their presurgery levels even after returning autologous blood (Figures 1b, 1c, 1e, 1f). Conversely, even though the clotting times in the EX-test and PT were prolonged after protamine antagonization, they returned to their presurgery levels at the end of the surgery (Figure 1d, 1g). Clot formation was well in the operative field, the field was dry, and there were no hemostasis problems. The operation, CPB, and aortic cross-clamping times were 323 min, 204 min, and 152 min, respectively. The intraoperative blood loss was 684 g, and the fluid balance was +2,380 mL.

The patient was transferred to the intensive care unit (ICU) under mechanical ventilation. The intensivist judged that there were no problems with breathing, circulation, or hemostasis, and the patient was extubated five hours after ICU admission. The total chest tube drainage volume was 360 mL for the first 12 hours after surgery, and the Hb level on postoperative day (POD) 1 was 10.4 g/dL. The patient was transferred to the general ward on POD 1 and discharged on POD 17 after rehabilitation and adjustments of diuretics and anticoagulants. There were no perioperative complications or allogeneic transfusions during her course.

## Discussion

With sufficient preoperative hematopoietic intervention and intraoperative ANH, we could maintain the perioperative Hb level in a JW patient who underwent combined cardiac surgery under CPB while avoiding allogeneic red blood cell (RBC) transfusion. ANH effectively reduces allogenic blood transfusions and blood loss because autologous blood drawn for ANH is rich in RBCs, platelets, fibrinogen, and all coagulation factors. Recent systematic reviews and meta-analyses of randomized trials suggest that ANH significantly reduces RBC transfusion and postoperative blood loss during cardiac surgery [5], as well as RBC transfusion during CABG [6].

Multiple factors, such as the preoperative Hb level, blood volume, autologous blood volume, hemodilution during drawing, and timing of returning autologous blood, affect ANH efficacy [7]. Among these factors, ANH efficacy is highly dependent on the ANH volume. Compared with ANH volumes <400 mL or 400-799 mL, larger ANH volumes (≥800 mL) were reportedly the most effective in reducing blood transfusions [8]. Additionally, ANH volumes of 5-8 mL/kg were not effective in reducing blood transfusions and postoperative blood loss, indicating that maximizing the ANH volume is essential for effective ANH [9]. Conversely, excessive ANH volumes can lead to anemia, especially during CPB, because of hemodilution by priming volume and blood consumption by CPB. Hb levels of <8 g/dL increase mortality and complication rates in cardiac surgery [10]. Furthermore, compared with a liberal RBC transfusion threshold (Hb <9.5 g/dL), a restrictive threshold (Hb <7 g/dL) during CPB was reportedly more prone to adverse events [11]. Similarly, hematocrit <22% during CPB was reportedly associated with acute kidney injury, neurological dysfunction, and mortality [4,12]. Therefore, we set the following lower limits for Hb: 7 g/dL during CPB and 7.5-8.0 g/dL immediately after CPB induction (considering the Hb depletion during CPB). Our observations show that the calculated ANH volume was adequate for maintaining appropriate Hb levels.

Preoperative Hb levels are also crucial for ANH efficacy. In cardiac surgery, preoperative erythropoietin administration reduces postoperative blood transfusion requirements [13]. Additionally, combination therapy with erythropoietin, iron, and vitamin B12 before cardiac surgery is effective in hematopoiesis [14]. In this case, preoperative administration of erythropoietin, iron, and vitamin B12 was effective and sufficient for increasing the Hb level to 14 g/dL (i.e., beyond the recommended preoperative level of 12 g/dL) [2].

Several studies have reported the efficacy of ANH against coagulation. For example, ANH reportedly improved the aPTT and fibrinogen levels slightly in cardiac surgery under CPB [3]. In thoracic aortic surgery, ANH improved the PT-INR, platelet count, and fibrinogen levels and reduced the transfusion of fresh frozen plasma and platelet concentrate [15]. Another study demonstrated the hemostatic effect of ANH on cardiac surgery under CPB [16]. Generally, the target platelets and fibrinogen levels in cardiac surgery are >5-10×10^4^/μL and >150-200 mg/dL, respectively [17,18]. In the present case, we could maintain these levels using an appropriate volume of ANH.

There are a few reports about the efficacy of ANH for JW patients undergoing simple cardiovascular surgery under CPB [19,20]. However, this is the first report on the effectiveness of ANH for JW in combined cardiac surgery under CPB with higher transfusion risk and the first to mention the importance of appropriate volumes for ANH considering hemodilution by CPB. However, as a limitation in case management, pump circuit selection may not have been optimized. Although common centrifugal pump circuits require lower priming volumes (approximately 600 mL) compared with roller pump circuits, a centrifugal pump was unavailable in our institution. Moreover, to minimize the amount of blood drawn, the use of pediatric blood collection tubes should have been considered.

## Conclusions

We succeeded in the perioperative management of a JW patient who underwent combined cardiac surgery under CPB. Preoperative hematopoiesis including erythropoietin and determining appropriate volumes considering the minimum Hb levels acceptable during CPB for intraoperative ANH were the critical factors in maintaining postoperative Hb levels and coagulation factors and safely achieving the religious belief of JW patients to avoid blood transfusion.

## References

[1] Salis S, Mazzanti VV, Merli G, Salvi L, Tedesco CC, Veglia F, Sisillo E (2008). Cardiopulmonary bypass duration is an independent predictor of morbidity and mortality after cardiac surgery. J Cardiothorac Vasc Anesth.

[2] Tanaka A, Ota T, Uriel N, Asfaw Z, Onsager D, Lonchyna VA, Jeevanandam V (2015). Cardiovascular surgery in Jehovah's Witness patients: the role of preoperative optimization. J Thorac Cardiovasc Surg.

[3] Smith BB, Nuttall GA, Mauermann WJ, Schroeder DR, Scott PD, Smith MM (2020). Coagulation test changes associated with acute normovolemic hemodilution in cardiac surgery. J Card Surg.

[4] Hwang NC (2015). Preventive strategies for minimizing hemodilution in the cardiac surgery patient during cardiopulmonary bypass. J Cardiothorac Vasc Anesth.

[5] Barile L, Fominskiy E, Di Tomasso N (2017). Acute normovolemic hemodilution reduces allogeneic red blood cell transfusion in cardiac surgery: a systematic review and meta-analysis of randomized trials. Anesth Analg.

[6] Li S, Liu Y, Zhu Y (2020). Effect of acute normovolemic hemodilution on coronary artery bypass grafting: a systematic review and meta-analysis of 22 randomized trials. Int J Surg.

[7] Murray D (2004). Acute normovolemic hemodilution. Eur Spine J.

[8] Goldberg J, Paugh TA, Dickinson TA (2015). Greater volume of acute normovolemic hemodilution may aid in reducing blood transfusions after cardiac surgery. Ann Thorac Surg.

[9] Casati V, Speziali G, D'Alessandro C, Cianchi C, Antonietta Grasso M, Spagnolo S, Sandrelli L (2002). Intraoperative low-volume acute normovolemic hemodilution in adult open-heart surgery. Anesthesiology.

[10] Hogervorst EK, Rosseel PM, van de Watering LM, Brand A, Bentala M, van der Bom JG, van der Meer NJ (2016). Intraoperative anemia and single red blood cell transfusion during cardiac surgery: an assessment of postoperative outcome including patients refusing blood transfusion. J Cardiothorac Vasc Anesth.

[11] Shehata N, Burns LA, Nathan H, Hebert P, Hare GM, Fergusson D, Mazer CD (2012). A randomized controlled pilot study of adherence to transfusion strategies in cardiac surgery. Transfusion.

[12] Karkouti K, Djaiani G, Borger MA (2005). Low hematocrit during cardiopulmonary bypass is associated with increased risk of perioperative stroke in cardiac surgery. Ann Thorac Surg.

[13] Yoo YC, Shim JK, Kim JC, Jo YY, Lee JH, Kwak YL (2011). Effect of single recombinant human erythropoietin injection on transfusion requirements in preoperatively anemic patients undergoing valvular heart surgery. Anesthesiology.

[14] Rössler J, Hegemann I, Schoenrath F (2020). Efficacy of quadruple treatment on different types of pre-operative anaemia: secondary analysis of a randomised controlled trial. Anaesthesia.

[15] Mladinov D, Eudailey KW, Padilla LA (2021). Effects of acute normovolemic hemodilution on post-cardiopulmonary bypass coagulation tests and allogeneic blood transfusion in thoracic aortic repair surgery: an observational cohort study. J Card Surg.

[16] Henderson RA, Judd M, Strauss ER, Gammie JS, Mazzeffi MA, Taylor BS, Tanaka KA (2021). Hematologic evaluation of intraoperative autologous blood collection and allogeneic transfusion in cardiac surgery. Transfusion.

[17] Boer C, Meesters MI, Milojevic M (2018). 2017 EACTS/EACTA Guidelines on patient blood management for adult cardiac surgery. J Cardiothorac Vasc Anesth.

[18] Kaufman RM, Djulbegovic B, Gernsheimer T (2015). Platelet transfusion: a clinical practice guideline from the AABB. Ann Intern Med.

[19] Kim SH, Yoon TG, Kim TY, Kim HK, Sung WS (2010). Cerebral oximetry monitoring during aortic arch aneurysm replacement surgery in Jehovah's Witness patient -a case report-. Korean J Anesthesiol.

[20] Obara S, Nakagawa M, Takahashi S, Akatu M, Isosu T, Murakawa M (2009). Anesthetic management for ascending aorta replacement in a patient who refused autologous transfusion for religious reasons. J Anesth.

